# Supplementary material for: Deep learning-based image analysis identifies a DAT-negative subpopulation of dopaminergic neurons in the lateral Substantia nigra
Source: Commun Biol. 2023 Nov 10;6:1146. doi: 10.1038/s42003-023-05441-6 (PMC10638391; doi:10.1038/s42003-023-05441-6)
Supplement: Supplementary file 2 — Supplementary Information [file 42003_2023_5441_MOESM2_ESM.pdf]

Supplementary information

Deep learning-based image-analysis identifies a DAT-negative subpopulation of dopaminergic neurons in the lateral Substantia nigra.

Burkert et al.

Supplementary Figures

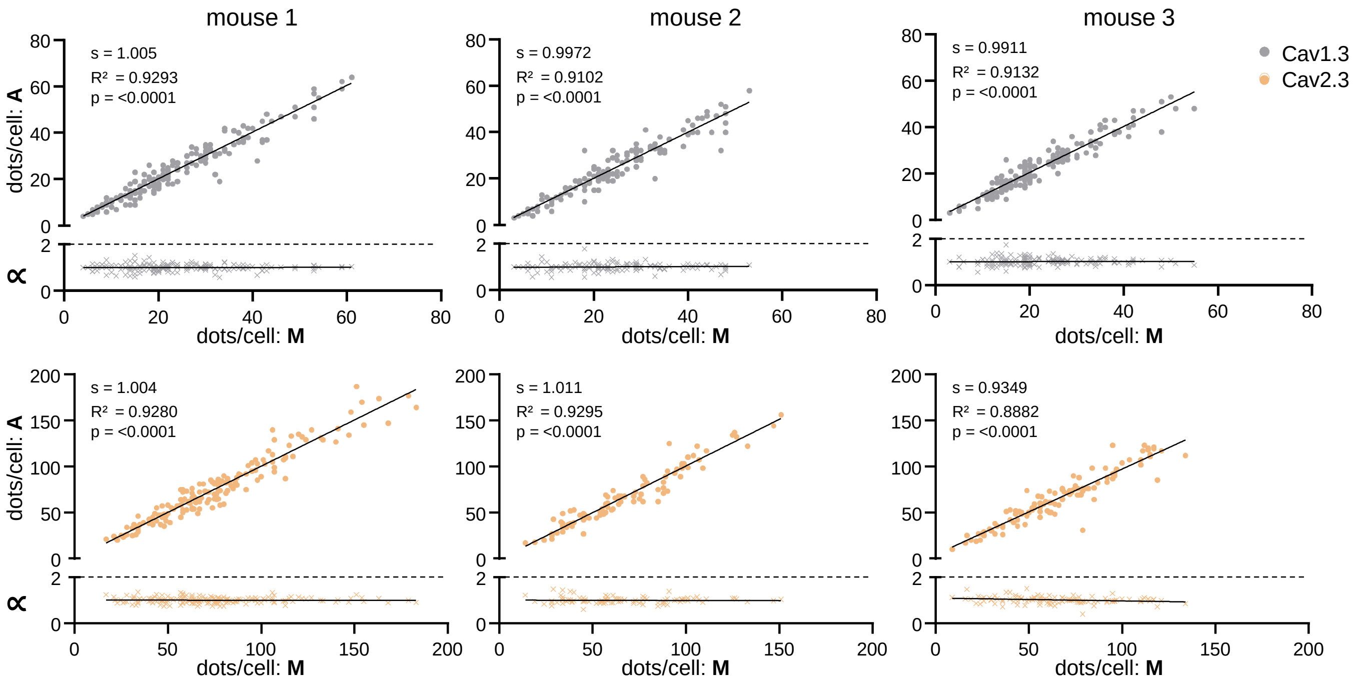

Figure S1: Comparison between manual and automated (DLAP-1) RNAscope-derived image analysis of mouse midbrain sections. Upper: single neuron correlations of Cav1.3 (grey) and Cav2.3 (light brown) mRNA-derived dot counts/TH-positive SN neuron between manual (M) and automated (A) analysis according to Pearson correlation test separately for each analysed mouse. Lower: corresponding proportionality constants ∝, calculated from the manual and automated dot count ratios.

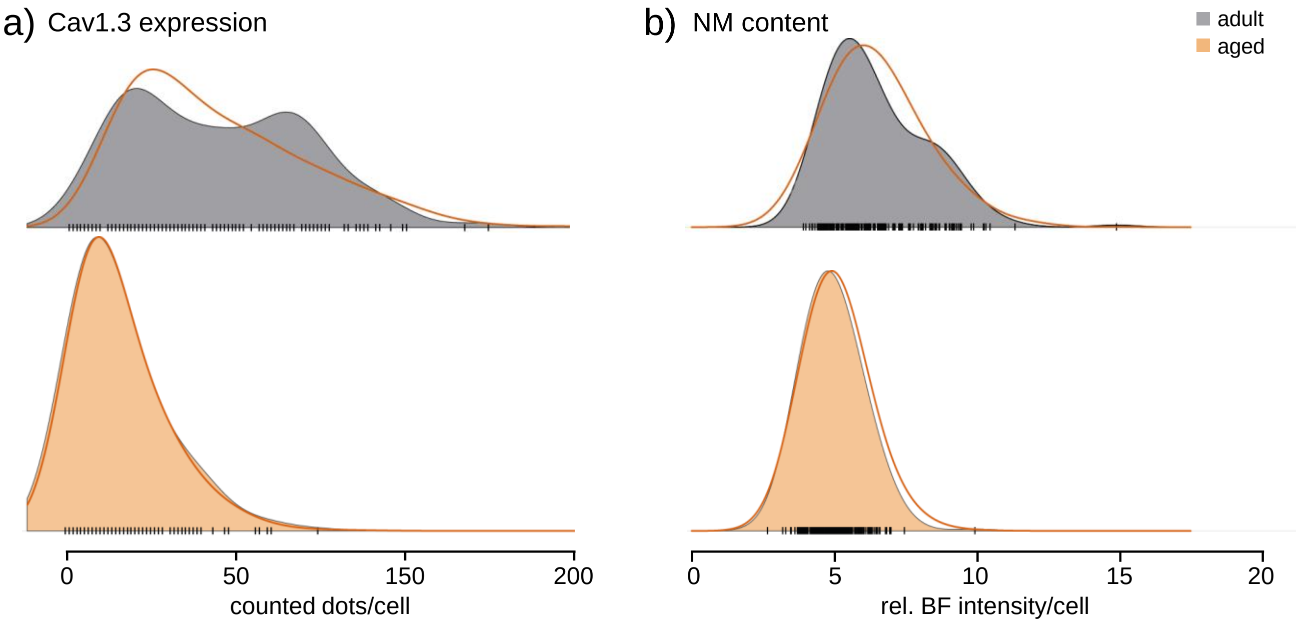

Figure S2: Posterior-predictive control of human RNAscope data model-fits. Cav1.3 expression (a) and NM content (BF light transmission, b) data is represented by vertical black lines and the colour-filled kernel densities for adult and aged brains, as indicated. Red continuous lines display the kernel distribution of data reproduced from the fitted model. Note the bimodal distribution in the group of adult brains in Cav1.3 expression and BF light transmission. The model does attribute for the wider base of the distribution of the number of detected Cav1.3 mRNA molecules (two components mixture of binomials) and BF light transmission (two components of beta), but does not represent the mixture proportion.

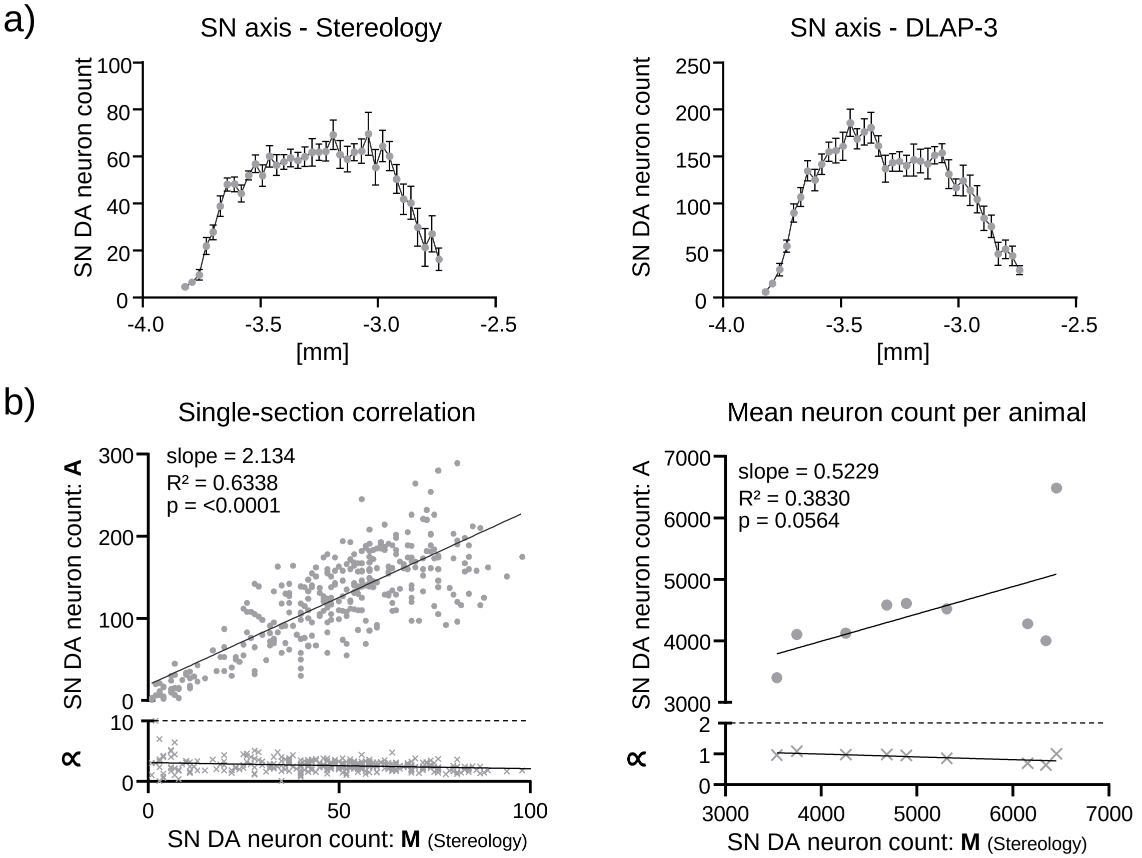

Figure S3: Comparison between unbiased stereology and automated (DLAP-3) IHC-derived image analysis of juvenile mouse midbrain sections. a) Mean SN DA neuron counts for all animals (N = 10) in all analysed sections along the caudal-rostral axis quantified via unbiased stereology (extrapolated via the optical fractionator method, M) or automatically (A) via DLAP-3. Data are given as mean ± SEM. b) Upper: correlations of individual SN DA neuron counts/section and animal (left) and mean SN DA neuron counts/animal (right) between unbiased stereology and automated analysis according to Pearson correlation test. Lower: corresponding proportionality constants ∝, calculated from the manual and automated cell counts ratios.

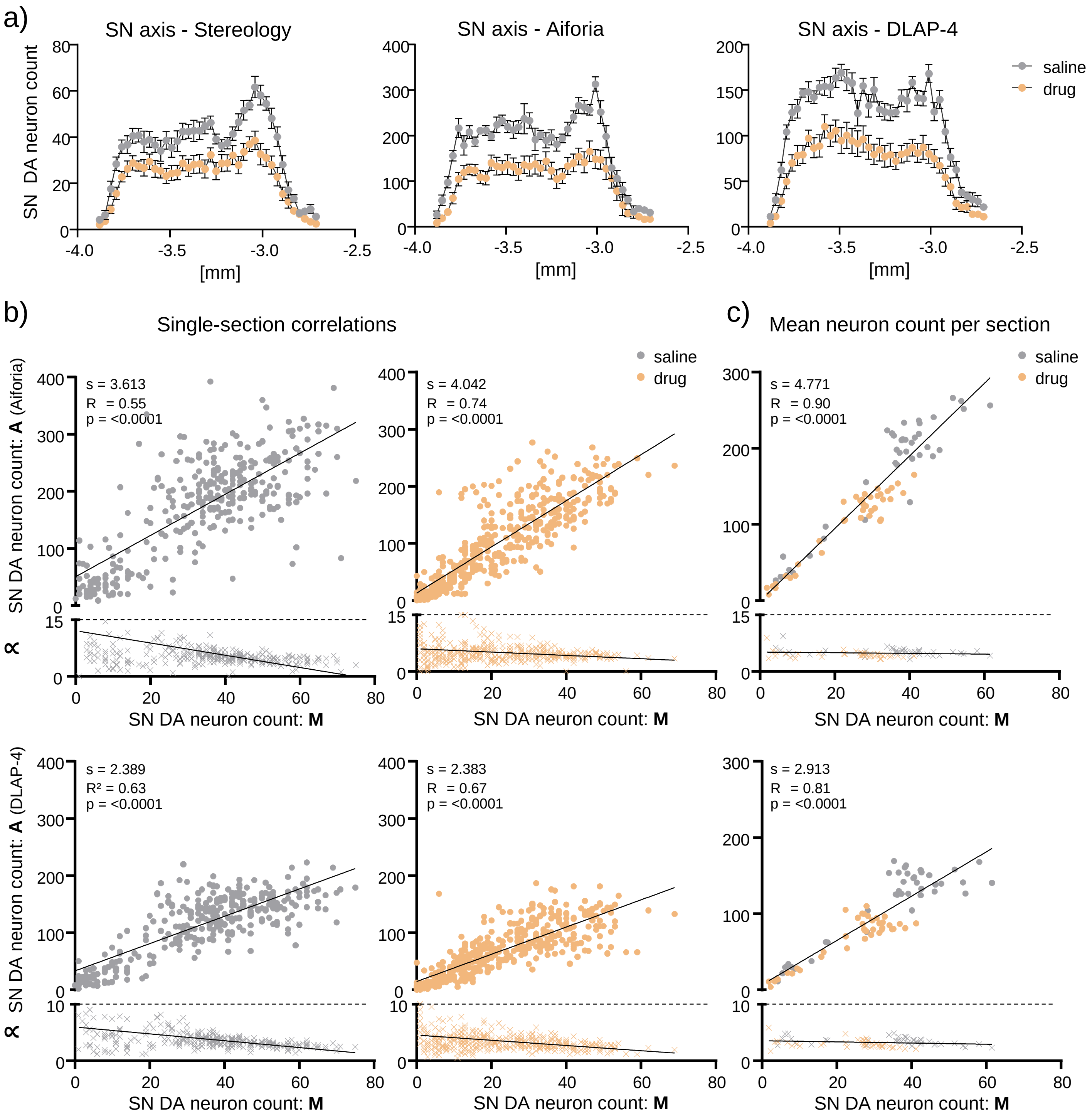

Figure S4: Comparison between unbiased stereology and automated (DLAP-4) IHC-derived image analysis of adult mouse midbrain sections. a) Mean SN DA neurons for all animals (saline: N = 9; neurodegenerative drug: N = 12) in all analysed sections along the caudal-rostral axis, quantified via unbiased stereology (extrapolated via the optical fractionator method, M) or automatically (A) via an algorithm provided by Aiforia, or via DLAP-4, as indicated. Data are given as mean ± SEM. b/c) Upper: correlations of individual SN DA neuron counts/section and animal (b) and mean animal counts/section along caudo-rostral axis (calculated from the individual values for each animal, c) between unbiased stereology and automated analysis (Aiforia, or DLAP-4, as indicated) according to Pearson correlation test. Lower: corresponding proportionality constants ∝, calculated from the manual and automated cell count ratios. Stereology data and Aiforia data modified from *Benkert et al., Nat. Commun. 2019*.

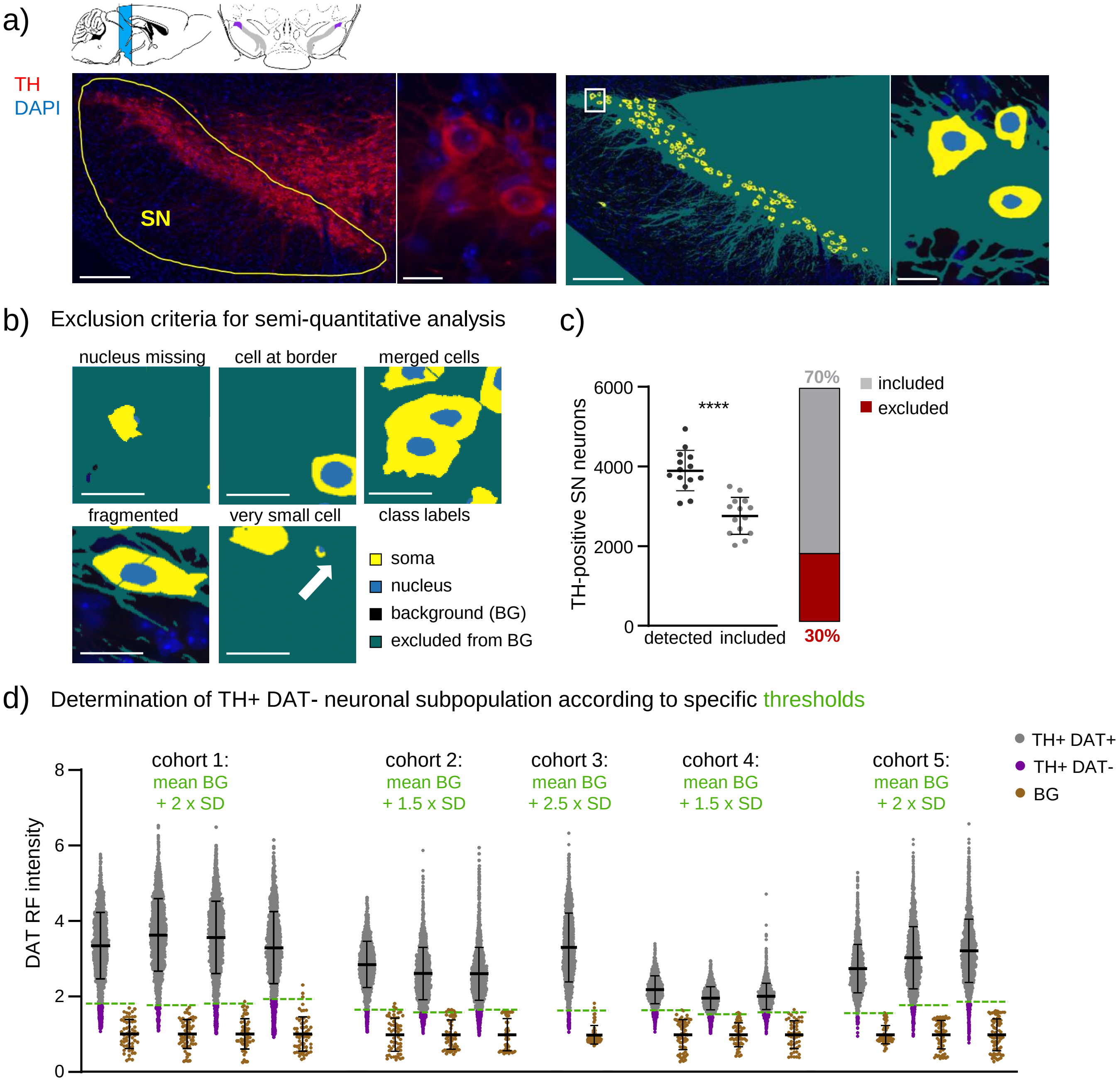

Figure S5: Relative DAT-signal quantification in TH-positive SN neurons from mouse midbrain sections with DLAP-5. a) Upper: exemplary sagittal and coronal mouse brain sections, modified from (Paxinos & Keith B. J. Franklin, 2007), illustrating the analysed caudo-rostral extent of the SN (bregma: -3.9 to -2.7, sagittal, blue), and its lateral parts in coronal sections (lateral SN: violet, non-lateral SN grey, compare Figure 7, S5). SN neurons after IF for TH (red). Nuclei are marked by DAPI (blue), the SN is delineated in yellow, the white box in the lateral SN indicates the position of DAT-negative neurons. Right: enlarged image of the white-boxed area from the left, output image after automated recognition of TH-labelled cell bodies (yellow area) and corresponding nuclei (blue area) with DLAP-5. Scale bars: 200 µm (left) and 20 µm (right). b) Examples of detected TH-positive neurons, where cell bodies were cropped (at the border), merged/not clearly separated from each other, fragmented, very small, or did not show a full nucleus. Such cells were excluded from relative quantification analysis. Scale bars: 20 µm. c) Left: numbers of TH-positive SN neurons/mouse, determined via DLAP-5. Shown are the numbers of all detected TH-positive SN neurons without any exclusion, according to the criteria in b), as well as the neuron numbers after exclusion-analysis, representing the neurons for which relative DAT-intensities were determined. Significant difference according to Mann-Whitney test (****: $p < 0.0001$). Right: percentage of excluded neurons. d) Plotted are the individual relative DAT fluorescence intensities (RF) for all included TH-positive SN neurons and for all backgrounds (BG, derived separately for each image, normalised to the mean BG in each animal), for each individual mouse, quantified via DLAP-5. The DAT-intensity thresholds for positive cells were determined individually for each mouse, according to the mean and the SD of the BG-signals, indicated as dashed green lines. Data are given as scatterplots and mean ± SD for all analysed mice (N = 14). All data are detailed in Table S13 and S14.

a) Illustration of analysed regions from the midbrain SN

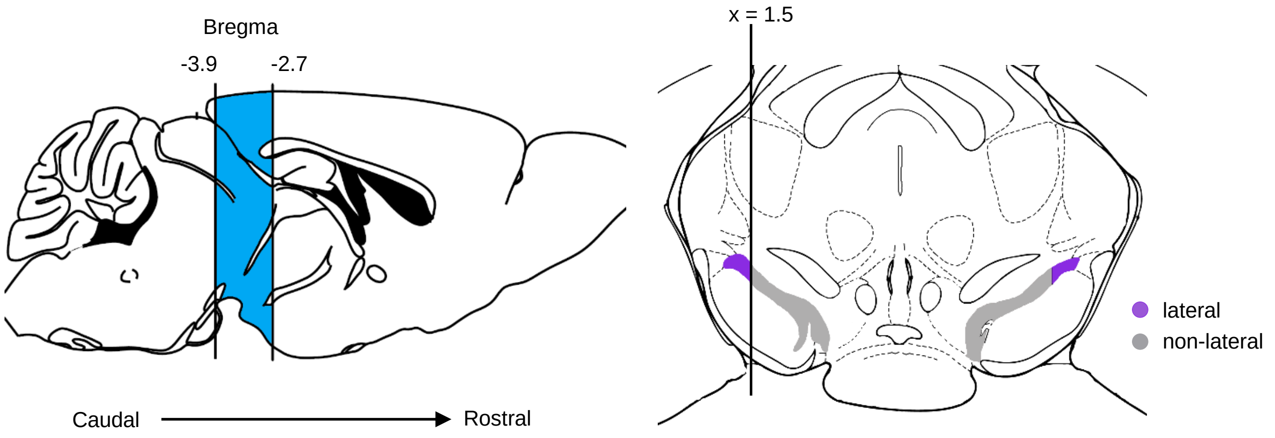

b) Defining the lateral SN subpopulation

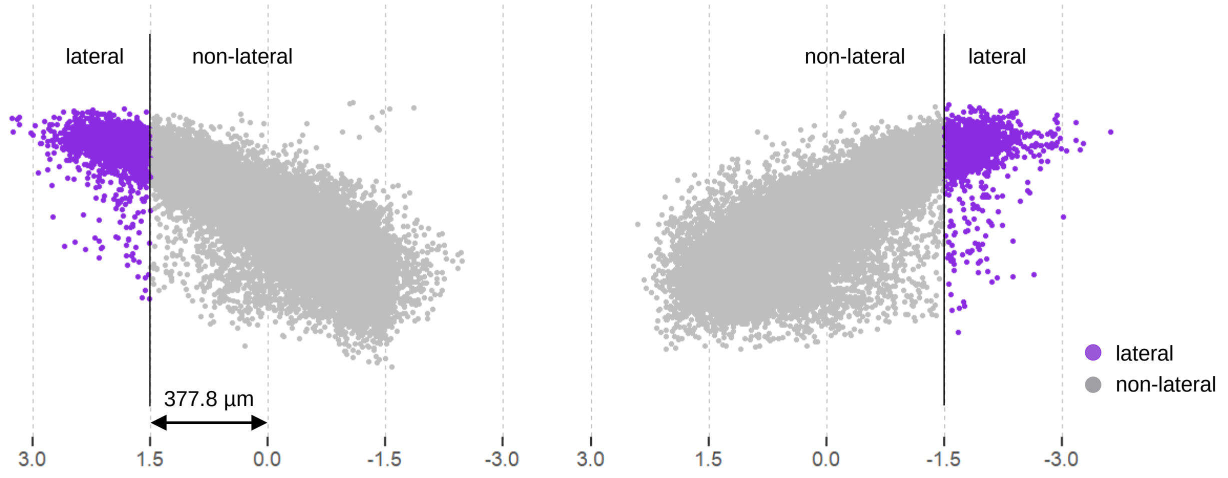

Figure S6: Definition of lateral and non-lateral SN-parts in coronal mouse brain sections, according to scaled x,y-coordinates. a) Sagittal (left) and coronal (right) mouse brain sections, modified from (Paxinos & Keith B. J. Franklin, 2007), illustrating the analysed caudo-rostral extent of the SN (bregma: -3.9 to -2.7, sagittal, blue), and its lateral parts in coronal sections (defined as >1.5 scaled x-units (>377.8 µm) lateral from each SN hemisphere-center; lateral SN: violet, non-lateral SN grey). Compare Figures 7-9. b) Plotted (here and in Figures 7-9) are the scaled x,y-coordinates for all TH-positive SN neurons that were further analysed for relative DAT signal intensities, from all analysed animals (N = 14). The lateral (violet) and the non-lateral (grey) SN are defined according to their scaled x,y-coordinates (determined via DLAP-5) and their distance (>1.5 x-units lateral) from the respective SN hemisphere-center (defined as 0.0 coordinates), visualized here by the vertical lines at 1.5/-1.5 (TH-positive neurons: lateral SN: left, n = 1749; right, n = 1547; non-lateral: left, n = 17693; right, n = 17515).

Online Supplement – S7

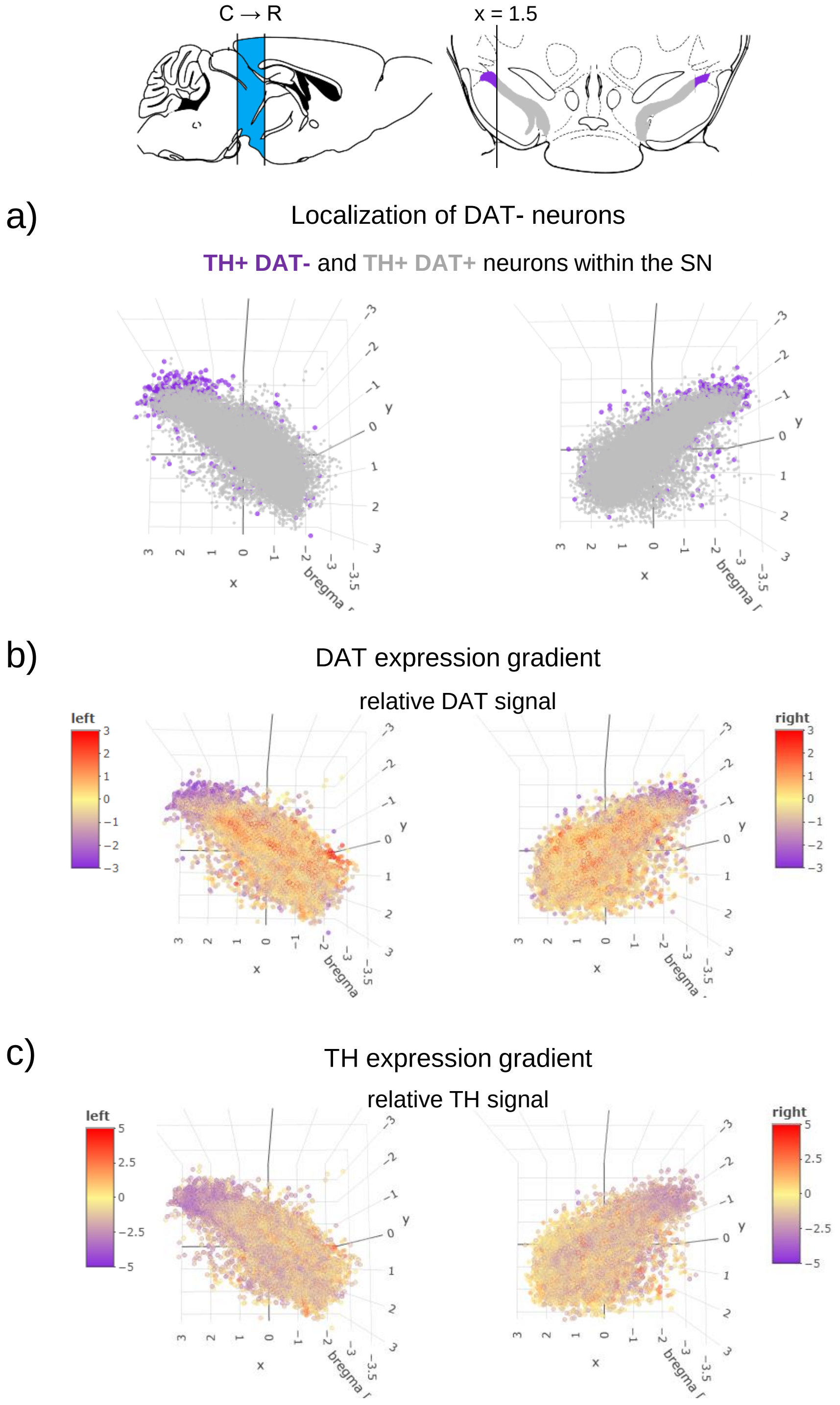

Figure S7: Thumbnail of the respective online interactive 3D-figure (HTML-file), plotting the anatomical location of all further analysed TH-positive SN neurons. a) Upper: sagittal (left) and coronal (right) mouse brain sections, modified from (Paxinos & Keith B. J. Franklin, 2007), illustrating the analysed caudo-rostral extent of the SN (bregma: -3.9 to -2.7, sagittal, blue), and its lateral parts in coronal sections (defined as >1.5 scaled x-units (>377.8 µm) lateral from each SN hemisphere-center (0,0); lateral SN: violet, non-lateral SN: grey, as in Figures 7-9, S6). Lower: plotted are the individual TH-positive SN neurons for all analysed animals (n = 38504, N = 14, as in Figure 7-9, S5), according to their scaled x,y,z-coordinates. The resulting anatomical 3D-representations display the medio-lateral distribution of the TH-positive DAT-negative SN neurons (violet) over the full rostro-caudal extent for all analysed mice. b/c) colour coded are the scaled relative DAT (b) and TH (c) signal intensities, of the individual TH-positive neurons from a), plotted according to their scaled x,y,z-coordinates. Colour coding for each neuron according to its individual deviation from the scaled mean signal-intensity (0.0) for each animal. The corresponding 2D maps are given in Figures 7-9. To access the interactive HTML-file, download it from the supplementary file - Figure S7.

a) Determination of TH+ D2- neuronal subpopulation

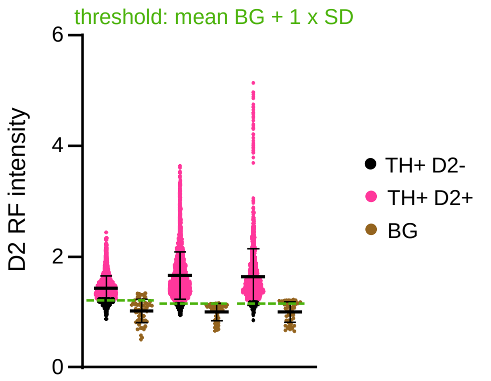

b) Determination of TH+ CB- neuronal subpopulation

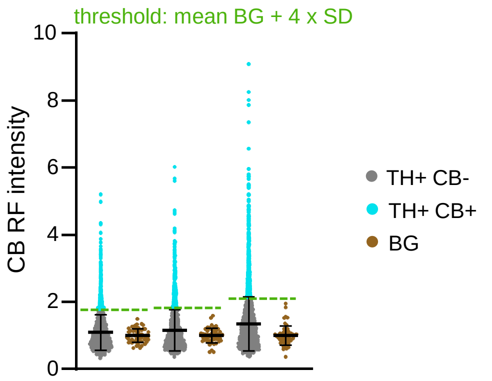

c) Determination of TH+ Aldh1A1- neuronal subpopulation

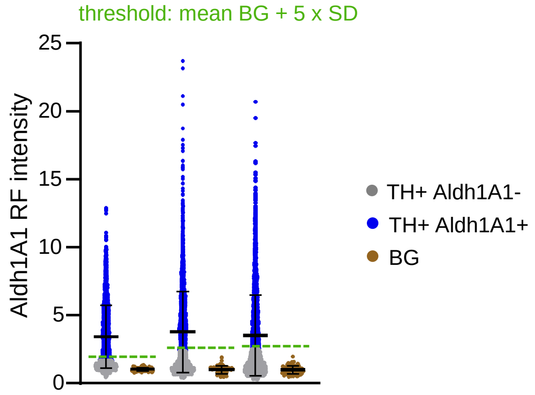

Figure S8: Relative D2, CB, and Aldh1A1-signal quantification in TH-positive SN neurons from mouse midbrain sections with DLAP-5. Plotted are the individual relative fluorescence intensities (RF) for a) D2, b) CB, and c) Aldh1A1, for all included TH-positive SN neurons and for all backgrounds (BG, derived separately for each image, normalised to the mean BG in each animal), for each individual mouse, quantified via DLAP-5. The respective intensity thresholds for D2-/CB-/Aldh1A1-positive cells were determined individually for each mouse, according to the mean and the SD of the BG-signals, and indicated as dashed green line. Data are given as scatterplots and mean ± SD for all analysed mice (N = 3; TH+ n = 2023-3417, BG n = 51-72) and detailed in Table S14.

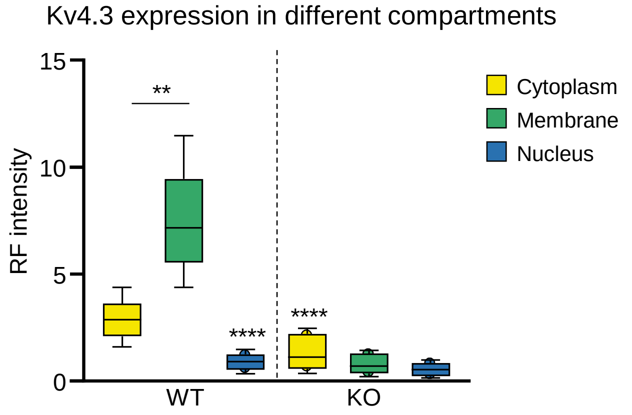

Figure S9: Automated (DLAP-6) IF-derived image analysis for cellular compartments of TH-positive SN neurons from WT and Kv4.3 KO mice. Kv4.3 relative fluorescence (RF) intensity/neuron in different sub-cellular compartments, quantified in WT and Kv4.3 KO mice, as indicated. Data are given as boxplots (median, 10-90 percentile) for all analysed neurons (WT: n = 179, Kv4.3 KO: n = 200). Significant differences according to Kruskal-Wallis with Dunn's multiple comparison tests (**: $p<0.01$, ****: $p < 0.0001$).

Supplementary tables

Table S1: Comparison of FCN and Deeplab3 network performances in distinct tasks. Performances were determined by comparing analysis results the test data to the ground truth data (provided by manual labeling). The following quality measures were assessed separately for each class: pixel error rate, rates for true positive (TP), true negative (TN), false positive (FP), false negative (FN) detection as well as specificity (spec.) [defined as TN/(TN+FP)] and sensitivity (sens.) [defined as TP/(TP+FN)].

D-LAP algorithm		Error [%]	class name	TP [%]	TN [%]	FP [%]	FN [%]	Spec [%]	Sens [%]
1	FCN	5.08	mRNA	0.3	98.9	0.7	0.0	99.3	92.5
			neuron	10.3	84.7	4.1	0.8	95.3	92.7
	DeepLab3	4.39	mRNA	0.0	99.7	0.0	0.3	100.0	0.0
			neuron	11.0	84.6	4.2	0.2	95.2	98.3
2 (human)	FCN	4.13	mRNA	0.2	98.9	0.9	0.0	99.1	94.7
			neuron	5.2	91.0	2.5	1.4	97.3	80.8
	DeepLab3	2.49	mRNA	0.0	99.8	0.0	0.2	100.0	0.0
			neuron	6.1	91.4	2.1	0.4	97.8	95.6
3	FCN	0.64	nucleus	0.0	99.4	0.0	0.6	100.0	1.7
	DeepLab3	0.61	nucleus	0.5	98.9	0.5	0.1	99.5	85.5
4	FCN	0.31	nucleus	0.2	99.5	0.3	0.0	99.7	46.7
	DeepLab3	0.26	nucleus	0.1	99.7	0.2	0.0	99.8	59.8
5	FCN	7.08	nucleus	1.0	97.3	1.5	0.2	98.4	80.9
			cytoplasm	3.7	90.8	4.9	0.7	94.8	83.8
	DeepLab3	3.3	nucleus	0.9	98.5	0.3	0.3	99.7	74.5
			cytoplasm	4.1	92.9	2.2	0.8	97.7	83.7
6	FCN	8.34	nucleus	9.4	87.9	1.2	1.5	98.7	87.2
			cytoplasm	12.4	82.8	4.1	0.8	95.3	93.4
			membrane	2.3	94.2	1.7	1.7	98.2	58.9
	DeepLab3	5.49	nucleus	3.0	93.5	2.5	1.0	97.4	75.6
			cytoplasm	9.7	89.0	0.8	0.5	99.1	95.0
			membrane	11.7	85.2	2.2	0.9	97.5	92.4

Table S2: Details of RNAscope probes. RNAscope target probes were obtained from the Advanced Cell Diagnostics (ACD) library. All probes detected but did not discriminate between known splice variants of the respective target genes. Abbreviations: NCBI: National Center for Biotechnology Information.

Species	Gene	ACD Cat No.	Assay target region according to NCBI Genbank gene accession no. (NM)
mouse	Tyrosine hydroxylase (*Th*)	317621	483 – 1603 of NM_009377.1 (exon 3-13)
	Cav1.3 (*Cacna1d*)	502591	522 – 1994 of NM_001302637.1 (exon 1-10)
	Cav2.3 (*Cacna1e*)	449211	2319 – 3317 of NM_009782.3 (exon 20-22)
human	Tyrosine hydroxylase (*TH*)	441651	596 – 1681 of NM_199292.2 (exon 4-14)
	Cav1.3 (*CACNA1D*)	502591	522 – 1994 of NM_001302637.1 (exon 1-10)

Table S3: Mean mRNA molecule numbers in individual mouse SN DA neurons, determined manually and via algorithm DLAP-1. Data and statistics for graphs in Fig. 3b. Number of total analysed neurons is given by n, N represents the number of analysed mice. P-values according to Two-way ANOVA followed by Tukey's multiple comparison (significant values in bold).

		manual				automated				p-value
	n<br>N	median	10-90 %	mean	±SD	median	10-90 %	mean	±SD	
Cav1.3	449	22	9-41	24	12	23	9-41	24	12	0.9988
	3	24	22-24	24	1	24	23-24	24	1	
Cav2.3	372	66	32-113	70	31	65	31-117	70	32/	0.9995
	3	67	66-75	69	5	67	65-75	69	5	
	Cav1.3 M vs Cav2.3 M, **<0.0001**; Cav1.3 A vs Cav2.3 A, **<0.0001**									

Table S4: Single cell correlation analyses between manually and DLAP-1 determined results. Data and statistics for graphs in Fig. 3c and S1a, according to Pearson correlation (slope of the linear regression with its own 95% confidence interval).

	Cav1.3	Cav2.3
all mice (N = 3)		
Pearson r (95% confidence interval)	0.959 (0.950-0.966)	0.959 (0.950-0.967)
R squared	0.919	0.920
p-value	<0.0001	<0.0001
No. of XY pairs	444	372
Slope (95% confidence interval)	0.998 (0.971-1.026)	0.989 (0.960-1.020)
Proportionality constant ∝	1.01 ± 0.16	1.00 ± 0.14
mouse 1		
Pearson r (95% confidence interval)	0.964 (0.952-0.973)	0.963 (0.951-0.973)
R squared	0.929	0.928
p-value	<0.0001	<0.0001
No. of XY pairs	168	167
Slope (95% confidence interval)	1.005 (0.962-1.047)	1.004 (0.961-1.047)
Proportionality constant ∝	1.01 ± 0.15	1.00 ± 0.13
mouse 2		
Pearson r (95% confidence interval)	0.954 (0.936-0.967)	0.964 (0.947-0.976)
R squared	0.910	0.930
p-value	<0.0001	<0.0001
No. of XY pairs	131	96
Slope (95% confidence interval)	0.997 (0.943-1.052)	1.011 (0.954-1.068)
Proportionality constant ∝	1.00 ± 0.17	1.00 ± 0.14
mouse 3		
Pearson r (95% confidence interval)	0.956 (0.939-0.968)	0.943 (0.917-0.960)
R squared	0.913	0.888
p-value	<0.0001	<0.0001
No. of XY pairs	145	109
Slope (95% confidence interval)	0.991 (0.941-1.042)	0.935 (0.871-0.998)
Proportionality constant ∝	1.02 ± 0.16	1.01 ± 0.15

Table S5: Details of human *post mortem* midbrain samples. Samples were provided by the German Brain Bank. The mean age of the aged cohort was significantly higher compared to that of the adult cohort (**0.0012; t-test), the mean RIN was significantly lower (*0.038; t-test), PMI was not significantly different. Abbreviations: AD: Alzheimer's disease; CERAD: Consortium to establish a registry for Alzheimer's disease; f: female; LB: Lewy Body; m: male; NFT: neurofibrillary tangles; PD: Parkinson's disease; PMI: *post mortem* interval, RIN: RNA integrity number.

Cohort	Age, sex	PMI [h]	RIN	Braak AD/NFT, PD	CERA-stage	LB-pathology
adult	46, f	27	8.5	- , 0	0	no
	34, m	10	8.3	- , 0	0	no
	46, m	19	8.4	- , 0	0	no
mean±SD	42 ± 7**	19 ± 9	8.4 ± 0.1*			
aged	76, m	24	6.9	I, -	A	no
	76, f	11	6.4	0, -	0	no
	81, f	18	4.0	II, -	0	no
mean±SD	78 ± 3	18 ± 7	5.8 ± 1.6			

Table S6: mRNA molecule numbers per human SN DA neuron, determined manually and via DLAP-2. Data and statistics for graphs in Fig. 4b and c. Number of total analysed neurons is given by n, N represents the number of analysed brain samples. P-values according to Two-way ANOVA followed by Tukey's multiple comparison (significant values in bold).

Cav1.3 mRNA-derived dots/cell										
		manual				automated				p-value
	n<br>N	median	10-90 %	mean	±SD	median	10-90 %	mean	±SD	
adult	223	36	11-68	39	23	37	13-78	42	25	0.6376
	3	38	34-43	38	5	39	34-47	40	7	
aged	258	10	2-32	13	12	12	2-38	17	15	0.2982
	3	16	10-16	14	3	19	13-20	17	4	
	adult M vs aged M, **<0.0001**; adult A vs aged A, **<0.0001**									
rel. BF signal intensity/cell										
		manual				automated				p-value
	n<br>N	median	10-90 %	mean	±SD	median	10-90 %	mean	±SD	
adult	223	16.4	10.8-22.3	16.4	4.5	16.7	11.0-23.1	16.7	4.7	0.9964
	3	16.2	14.5-18.9	16.5	2.2	16.6	14.7-19.0	16.7	2.2	
aged	258	20.4	15.4-26.8	21.2	5.4	20.7	15.6-26.5	21.1	5.1	>0.9999
	3	21.6	20.7-22.2	21.5	0.8	21.5	20.6-22.2	21.4	0.8	
	adult M vs aged M, **<0.0001**; adult A vs aged A, **<0.0001**									
BF-adjusted Cav1.3 mRNA-derived dots/cell										
		manual				automated				p-value
	n<br>N	median	10-90 %	mean	±SD	median	10-90 %	mean	±SD	
adult	180	31	18-50	32	12	31	17-49	32	12	>0.9999
	3	33	30-34	32	2	33	29-34	32	3	
aged	224	17	9-30	19	9	20	11-35	21	10	**0.0436**
	3	18	14-21	17	3	21	16-22	20	3	
	adult M vs aged M, **<0.0001**; adult A vs aged A, **<0.0001**									

Table S7: Single cell correlation analyses between manually and DLAP-2 determined results. Data and statistics for graphs in Fig. 4d and S1b and c, according to Pearson correlation (slope of the linear regression with its own 95% confidence interval).

	Cav1.3 mRNA dot counts/cell	rel. BF intensity/cell	Cav1.3 molecules, NM (BF)-adjusted according to model
all human samples (N = 6)			
Pearson r (95% confidence interval)	0.964 (0.957-0.970)	0.992 (0.990-0.993)	0.897 (0.877-0.915)
R squared	0.928	0.983	0.805
p-value	<0.0001	<0.0001	<0.0001
No. of XY pairs	481	481	404
Slope (95% confidence interval)	1.034 (1.008-1.060)	0.968 (0.957-0.979)	0.867 (0.827-0.910)
Proportionality constant ∝	1.28 ± 1.18	1.01 ± 0.03	1.10 ± 0.25
adult samples (N = 3)			
Pearson r (95% confidence interval)	0.975 (0.967-0.980)	0.996 (0.995-0.997)	0.876 (0.837-0.906)
R squared	0.950	0.992	0.767
p-value	<0.0001	<0.0001	<0.0001
No. of XY pairs	223	223	180
Slope (95% confidence interval)	1.044 (1.012-1.076)	1.033 (1.021-1.046)	0.851 (0.781-0.920)
Proportionality constant ∝	1.23 ± 1.51	1.01 ± 0.02	1.01 ± 0.18
aged samples (N = 3)			
Pearson r (95% confidence interval)	0.890 (0.861-0.913)	0.988 (0.984-0.990)	0.872 (0.836-0.900)
R squared	0.792	0.976	0.872
p-value	<0.0001	<0.0001	<0.0001
No. of XY pairs	258	258	224
Slope (95% confidence interval)	1.130 (1.059-1.201)	0.940 (0.9221-0.958)	0.977 (0.905-1.050)
Proportionality constant ∝	1.33 ± 0.78	1.00 ± 0.03	1.17 ± 0.27

Table S8: SN DA neuron numbers determined via unbiased stereology and via DLAP-3 on DAB-stained sections. Data and statistics for graphs in Fig. 5b. Given are the numbers of all TH-positive SN neurons, unilaterally determined for each mouse by stereology or algorithm. N represents the number of analysed mice. P-values according to Mann-Whitney test (significant values in bold).

		manual (Stereology)				automated			
	N	median	10-90 %	mean	±SD	median	10-90 %	mean	±SD
	10	4789	3561-6444	4951	1079	4199	3100-6297	4317	911.8
p-value	Mann-Whitney test: 0.2176								

Table S9: SN DA neuron numbers determined via unbiased stereology and via DLAP-4 on DAB- and hematoxylin-stained sections. Data and statistics for graphs in Fig. 6b. Given are the numbers of all TH-positive SN neurons/mouse, unilaterally determined by stereology or algorithm. N represents the number of analysed mice. P-values according to Two-way ANOVA followed by Tukey's multiple comparison (significant values in bold).

	saline					PD-drug					p-value
	N	median	10-90 %	mean	±SD	N	median	10-90 %	mean	±SD	
manual (stereology)	9	3826	2666-4919	3959	643	12	2556	1209-3587	2504	922	**0.022**
automated (Aiforia)		6881	5749-8418	7048	823		4619	1680-5913	4108	1596	**<0.0001**
automated (Wolution)		4797	3442-5084	4614	500		2768	1314-4230	2708	975	**0.0010**
p-values	saline M vs. saline A (W), 0.7441; drug M vs. drug A (W), 0.9963; saline M vs. saline A (A), **<0.0001**; drug M vs. drug A (A), **0.0036**; saline A (W) vs saline A (A), <**0.0001**; drug A (W) vs drug A (A), **0.0156**										

Table S10: Remaining SN DA neurons after neurodegenerative drug treatment, determined via unbiased stereology and via DLAP-4. Data and statistics for graphs in Fig. 6b. Given are the percentages of remaining TH-positive SN neurons/mouse after drug treatment relative to saline, unilaterally determined by stereology or algorithm. N represents the number of analysed mice. P-values according to Kruskal-Wallis with Dunn's multiple comparison (no significant differences).

group	N	median	10-90 %	mean	±SD
manual (Stereology)	12	64.6	30.5-90.6	63.3	23.3
automated (Aiforia)		65.5	23.8-83.9	58.3	22.7
automated (Wolution)		60.0	28.5-91.7	58.7	21.1
p-values	M vs A (W), >0.9999; M vs A (A), >0.9999, A (W) vs A (A), >0.9999				

Table S11: Single cell correlation analyses between manually and DLAP-3 and DLAP-4 determined results. Data and statistics for graphs in Fig. 5c/6c, according to Pearson correlation (slope of the linear regression with 95% confidence interval).

	juvenile	adult (Aiforia)	adult (Wolution)
Individual sections			
Pearson r (95% confidence interval)	0.796 (0.753-0.832)	0.742 (0.688-0.787)	0.791 (0.746-0.829)
R squared	0.634	0.5500	0.626
p-value	<0.0001	<0.0001	<0.0001
No. of XY pairs	332	3189	319
Slope (95% confidence interval)	2.134 (1.959-2.310)	3.613 (3.251-3.974)	2.389 (2.185-2.593)
Proportionality constant ∝	2.60 ± 1.00	6.48 ± 8.43	3.86 ± 2.34
**Individual sections: PD-drug**			
Pearson r (95% confidence interval)		0.860 (0.833-0.882)	0.815 (0.781-0.845)
R squared		0.739	0.665
p-value		<0.0001	<0.0001

No. of XY pairs		433	433
Slope (95% confidence interval)		4.042 (3.814-4.269)	2.383 (2.223-2.544)
Proportionality constant ∝		5.00 ± 3.16	3.46 ± 2.12
Mean (for all animals) per section			
Pearson r (95% confidence interval)	0.889 (0.828-0.929)	0.946 (0.917-0.965)	0.897 (0.844-0.933)
R squared	0.790	0.895	0.805
p-value	<0.0001	<0.0001	<0.0001
No. of XY pairs	73	80	80
Slope (95% confidence interval)	2.383 (2.092-2.674)	4.771 (4.402-5.139)	2.913 (2.590-3.236)
Proportionality constant ∝	2.58 ± 0.58	4.81 ± 1.01	3.20 ± 0.74
Mean per animal			
Pearson r (95% confidence interval)	0.619 (-0.018-0.898)	0.901 (0.768-0.960)	0.919 (0.808-0.967)
R squared	0.383	0.812	0.845
p-value	0.0564	<0.0001	<0.0001
No. of XY pairs	10	21	21
Slope (95% confidence interval)	0.5229 (-0.018-1.064)	1.639 (1.260-2.018)	1.057 (0.8396-1.275)
Proportionality constant ∝	0.89 ± 0.15	1.71 ± 0.29	1.13 ± 0.15

Table S12: Number of TH-positive SN neurons analysed via DLAP-5. Data and statistics for graphs in Fig. 7b, 8b and S5c. Given are the numbers of all TH-positive SN neurons/mouse, bilaterally determined by the algorithm, the number of neurons excluded/included neurons for further DAT analysis (criteria specified in Figure S5b), and the determined number of TH positive DAT positive/negative SN neurons. Rostral, medial and caudal locations were determined according to the respective stereotaxic coordinates (bregma; rostral: -2.7 to -3.1 mm; medial: -3.1 to -3.5; caudal: -3.5 to -3.9). Lateral and non-lateral SN-areas were defined as detailed in Figure S6. N represents the number of analysed mice. P-values according to Wilcoxon or Mann-Whitney tests (significant values in bold).

	N	mean	±SD	%	mean	±SD	%	mean	±SD	%
		all detected TH+ SN neurons			excluded TH+ neurons from further DAT analysis			included TH+ neurons for further DAT analysis		
whole SN	14	3902	507	100	1151	220	30 ± 6	2750	462	70 ± 6
p-value		MWU: all vs analysed TH+ neurons **<0.0001**								
		identified TH+ DAT+			identified TH+ DAT-			all included TH+ neurons		
whole SN	14	2633	442	96 ± 2	118	50	4 ± 2	2750	462	100
rostral		876	176	99 ± 1	10	8	1 ± 1	886	178	100
medial		987	248	95 ± 2	53	24	5 ± 2	1040	260	100
caudal		767	126	93 ± 3	57	25	7 ± 3	824	132	100
p-value		Wilcoxon: all analysed TH+ vs analysed TH+ DAT+ **0.0001**<br>Chi-square: rostral vs medial **<0.0001**, rostral vs caudal **<0.0001**, caudal vs medial **<0.0001**								
**lateral SN**		identified TH+ DAT+			identified TH+ DAT-			all included TH+ neurons		
whole axis	14	194	27	83 ± 6	41	19	17 ± 6	235	41	100
rostral		56	19	95 ± 5	4	5	5 ± 5	60	22	100
medial		104	26	85 ± 5	19	9	15 ± 5	123	32	100
caudal		34	17	63 ± 15	19	9	37 ± 15	53	20	100

p-value		Wilcoxon: all analysed TH+ vs analysed TH+ DAT+ **0.0001**<br>Chi-square: rostral vs medial 0.1026, rostral vs caudal **0.0001**, caudal vs medial **0.0025**								
**non-lateral SN**		identified TH+ DAT+			identified TH+ DAT-			all included TH+ neurons		
whole axis	14	2436	405	97 ± 2	79	37	3 ± 2	2515	410	100
rostral		820	170	99 ± 1	7	5	1 ± 1	827	170	100
medial		883	219	96 ± 2	34	16	4 ± 2	917	223	100
caudal		733	118	95 ± 3	38	22	5 ± 3	771	122	100
p-value		Wilcoxon: all analysed TH+ vs analysed TH+ DAT+ **0.0001**<br>Chi-square: rostral vs medial **<0.0001**, rostral vs caudal **<0.0001**, caudal vs medial 0.2163								

Table S13: Relative DAT immunofluorescence signal intensities (RF) for included TH-positive SN neurons, for each mouse, determined via DLAP-5. Data and statistics for graphs in Fig. S5d, and S7 (background normalised). n represents the number of total analysed TH-positive SN neurons or the number background (BG) images. TH-positive neurons with DAT-signals over the respective “threshold”-values were defined as DAT-positive.

	**RF included-TH-positive SN neurons**					**RF Background (BG)**					**RF threshold**
mouse No	n	median	10-90 %	mean	±SD	n	median	10-90 %	mean	±SD	mean BG +2SD
1	3407	3.4	2.2-4.5	3.3	0.9	56	1.0	0.5-1.5	1.0	0.4	1.8
2	2945	3.6	2.4-4.9	3.6	1.0	58	0.9	0.5-1.4	1.0	0.4	1.8
3	2969	3.6	2.3-4.8	3.6	1.0	56	0.9	0.5-1.6	1.0	0.4	1.8
4	3129	3.4	2.1-4.7	3.4	1.0	60	0.9	0.5-1.6	1.0	0.5	1.9
											mean BG +1.5SD
5	2715	2.9	2.1-3.7	2.9	0.6	58	1.1	0.4-1.6	1.0	0.4	1.7
6	2314	2.6	1.8-3.6	2.6	0.7	54	0.9	0.6-1.6	1.0	0.4	1.6
7	3417	2.6	1.8-3.5	2.6	0.7	54	0.9	0.5-1.6	1.0	0.4	1.6
											mean BG +2.5SD
8	2665	3.3	2.2-4.7	3.4	0.9	56	0.9	0.8-1.5	1.0	0.3	1.6
											mean BG +1.5SD
9	2132	2.2	1.7-2.7	2.2	0.4	58	1.0	0.4-1.4	1.0	0.4	1.6
10	2330	2.0	1.6-2.4	2.0	0.3	55	0.9	0.5-1.5	1.0	0.3	1.5
11	2023	2.0	1.6-2.5	2.0	0.4	51	1.0	0.4-1.5	1.0	0.4	1.6
											mean BG +2SD
12	2329	2.7	2.0-3.7	2.8	0.7	72	0.9	0.8-1.5	1.0	0.2	1.5
13	2994	3.0	2.1-4.2	3.1	0.8	64	1.1	0.4-1.5	1.0	0.4	1.8
14	3135	3.2	2.3-4.4	3.3	0.9	68	0.9	0.4-1.6	1.0	0.4	1.9

Table S14: Relative D2, CB and Aldh1A1 immunofluorescence signal intensities (RF) in TH-positive SN neurons for each mouse, determined via DLAP-5. Data and statistics for graphs in Fig. 11 and S8 (background normalised). n represents the number of total analysed TH-positive neurons or the number background (BG) images, as indicated. TH-positive neurons with D2/CB/Aldh1A1-signals over the respective "threshold"-values were defined as immuno-positive for the respective protein.

	**RF TH-positive neurons**					**RF Background (BG)**					**RF threshold**
mouse No	n	median	10-90 %	mean	±SD	n	median	10-90 %	mean	±SD	
Dopamine D2 receptor (D2)											mean BG +1SD
9	2132	1.4	1.2-1.7	1.4	0.2	58	1.0	0.7-1.3	1.0	0.2	1.2
10	2330	1.6	1.2-2.2	1.7	0.4	55	1.1	0.7-1.1	1.0	0.2	1.2
11	2023	1.5	1.2-2.2	1.6	0.5	51	1.0	0.7-1.2	1.0	0.2	1.2
Calbindin-d28k (CB)											mean BG +4SD
5	2715	1.0	0.6-1.8	1.1	0.5	58	1.0	0.7-1.3	1.0	0.2	1.8
6	2314	1.0	0.6-1.9	1.2	0.6	54	1.0	0.8-1.3	1.0	0.2	1.9
7	3417	1.2	0.6-2.2	1.3	0.8	54	1.0	0.7-1.4	1.0	0.3	2.2
Aldehyde dehydrogenase 1A1 (Aldh1A1)											mean BG +5SD
12	2329	2.8	1.1-6.6	3.4	2.3	72	1.0	0.8-1.2	1.0	0.1	1.6
13	2994	3.0	0.9-7.8	3.8	3.0	64	1.0	0.6-1.3	1.0	0.3	2.4
14	3135	2.5	0.8-7.8	3.5	3.0	68	1.0	0.6-1.4	1.0	0.3	2.5

Table S15: Relative D2, CB and Aldh1A1 immunofluorescence signal intensities (RF) in DAT-positive and DAT-negative TH-positive SN neurons, determined via DLAP-5. Data and statistics for graphs in Fig. 10 (background normalised). Further processed data from table S14. n represents the number of total analysed TH-positive neurons. P-values according to Mann-Whitney tests (significant values in bold).

**marker gene**	**TH-positive SN neuron subtype**	**DAT-positive SN neuron subtype**			**DAT-negative SN neuron subtype**			p-value
		n	Mean RF	±SD	n	Mean RF	±SD	
**Dopamine D2-autoreceptor (D2)**	all TH-positives	6108	1.58	0.41	377	1.41	0.49	**<0.0001**
	D2-positive	5663	1.62	0.41	255	1.56	0.53	**0.0035**
	D2-negative	445	1.13	0.05	122	1.09	0.07	**<0.0001**
**Calbindin-d28k (CB)**	all TH-positives	8063	1.18	0.64	383	1.91	1.02	**<0.0001**
	CB-positive	722	2.63	0.89	143	2.96	0.83	**<0.0001**
	CB-negative	7341	1.03	0.39	240	1.28	0.42	**<0.0001**
**Aldehyde dehydrogenase (Aldh1A1)**	all TH-positive	8206	3.65	2.80	252	1.02	0.74	**<0.0001**
	Aldh1A1-positive	4810	5.31	2.57	14	3.55	0.80	**<0.0001**
	Aldh1A1-negatve	3396	1.30	0.47	238	0.87	0.37	**<0.0001**

Table S16: TH-positive SN neuron marker gene co-expression analysis. Data and statistics for graphs in Fig. 7b/d and 11. The total numbers of marker gene-positive and -negative neurons is represented by n. The relative % of marker gene and TH co-expression are given for all analysed TH-positive (TH+) SN neurons as well as separately for lateral (lat) and non-lateral (non-lat) TH+ neurons. P-values according to Chi-square test (significant values in bold).

**marker gene expression**	**all TH-positive SN neurons**			**lateral TH+ SN neurons**			**non-lateral TH+ SN neurons**			p-value DAT+ vs. DAT-
	n	Mean %	±SD	n	Mean %	±SD	n	Mean %	±SD	
TH+	38504	100	-	3296	8.6	0.7	35208	91.4	0.7	
TH+ DAT+	36854	95.7	1.7	2717	83.0	5.8	34103	96.8	1.6	
TH+ DAT-	1649	4.3	1.7	579	17.0	5.8	1105	3.2	1.6	
TH+ D2+	5908	91.0	5.5	543	89.7	7.4	5365	91.2	5.3	
TH+ D2-	577	9.0	5.5	62	10.3	7.4	515	8.8	5.3	
TH+ CB+	845	10.0	0.8	238	33.3	7.3	607	7.8	0.5	
TH+ CB-	7601	90.0	0.8	481	66.7	7.3	7120	92.2	0.5	
TH+ Aldh1A1+	4811	57.8	7.6	127	19.2	12.7	4684	61.3	7.2	
TH+ Aldh1A1-	3647	42.2	7.6	585	80.8	12.7	3062	38.7	7.2	
TH+ DAT+ D2+	5663	92.7	5.8	490	92.4	6.7	5156	92.6	4.7	all: **<0.0001**<br>lat: **<0.0001**<br>non-lat: **<0.0001**
TH+ DAT+ D2-	445	7.3	5.8	40	7.6	6.7	412	7.4	4.7	
TH+ DAT- D2+	255	66.9	23.0	53	69.7	14.0	209	66.0	19.4	
TH+ DAT- D2-	122	33.1	23.0	22	30.3	14.0	103	34.0	19.4	
p-value (lat vs. non-lat)				lat vs non-lat DAT+: 0.8625<br>lat vs non-lat DAT-: 0.5844						
TH+ DAT+ CB+	722	8.9	0.8	142	25.2	5.8	561	7.4	0.7	all: **<0.0001**<br>lat: **<0.0001**<br>non-lat: **<0.0001**
TH+ DAT+ CB-	7341	91.1	0.8	431	74.8	5.8	6917	92.6	0.7	
TH+ DAT- CB+	143	38.8	7.0	96	66.9	7.9	46	19.8	6.6	
TH+ DAT- CB-	240	61.2	7.0	50	33.1	7.9	203	80.2	6.6	
p-value (lat vs. non-lat)				lat vs non-lat DAT+: **<0.0001**<br>lat vs non-lat DAT-: **<0.0001**						
TH+ DAT+ Aldh1A1+	4810	59.4	8.8	124	21.5	12.4	4673	62.2	6.9	all: **<0.0001**<br>lat: **<0.0001**<br>non-lat: **<0.0001**
TH+ DAT+ Aldh1A1-	3396	40.6	8.8	473	78.5	12.4	2929	37.8	6.9	
TH+ DAT- Aldh1A1+	14	7.0	4.2	3	5.9	8.3	11	5.5	4.9	
TH+ DAT- Aldh1A1-	238	93.0	4.2	112	94.1	8.3	133	94.5	4.9	
p-value (lat vs. non-lat)				lat vs non-lat DAT+: **<0.0001**<br>lat vs non-lat DAT-: 0.0753						

Table S17: Soma size of all analysed TH-positive SN neurons, determined via DLAP-5. Data for graphs in Fig. 7e. Cell body sizes [μm^2] were estimated according to the TH-positive area that was marked by DLAP-5, for all analysed TH-positive neurons, and are given for different SN-neuron subtypes, defined according to their co-expression profile.

**marker gene co-expression type**	**analysed mice**	**all TH+ SN neurons cell body area [μm^2]**		**lateral TH+ SN neurons cell body area [μm^2]**		**non-lateral TH+ SN neurons cell body area [μm^2]**	
	N	Mean	±SD	Mean	±SD	mean	±SD
all TH+ DAT+	14	195.5	14.4	186.4	16.6	196.2	14.8
all TH+ DAT-	14	131.0	16.5	117.9	19.1	138.2	16.6
caudal TH+ DAT+	14	194.5	16.2	185.1	23.0	195.5	16.3
caudal TH+ DAT-	14	127.7	22.2	115.5	19.8	142.5	24.2
medial TH+ DAT+	14	189.5	15.3	197.0	18.8	187.4	16.0
medial TH+ DAT-	14	131.7	15.9	120.9	17.5	140.0	16.3
rostral TH+ DAT+	14	202.7	14.9	210.1	14.3	200.9	15.7
rostral TH+ DAT-	14	150.2	36.0	124.2	33.3	167.9	39.8
TH+ D2+	3	183.9	4.5	192.4	4.6	182.1	5.2
TH+ D2-	3	167.7	7.8	161.5	2.7	168.7	9.0
TH+ CB+	3	161.2	9.7	133.2	1.8	174.5	9.8
TH+ CB-	3	193.4	8.1	199.6	5.7	192.1	8.6
TH+ Aldh1A1+	3	176.0	5.6	189.6	3.0	174.8	6.3
TH+ Aldh1A1-	3	183.1	3.5	163.4	3.3	193.1	5.4
TH+ DAT+ D2+	3	185.9	3.8	198.7	6.2	183.3	4.9
TH+ DAT+ D2-	3	174.6	5.5	187.0	11.7	173.2	8.0
TH+ DAT- D2+	3	140.8	9.0	122.1	7.9	149.5	8.3
TH+ DAT- D2-	3	145.5	8.7	133.8	13.5	148.6	11.2
TH+ DAT+ CB+	3	172.7	6.6	158.2	2.6	176.8	7.3
TH+ DAT+ CB-	3	195.0	7.9	203.5	5.3	193.2	8.4
TH+ DAT- CB+	3	104.2	7.6	96.0	4.6	141.6	28.1
TH+ DAT- CB-	3	145.3	10.6	132.0	8.8	151.3	13.8
TH+ DAT+ Aldh1A1+	3	176.1	5.5	189.3	3.1	175.0	6.2
TH+ DAT+ Aldh1A1-	3	187.6	5.0	171.8	5.2	194.8	5.9
TH+ DAT- Aldh1A1+	3	132.6	27.3	263.9	0.0	126.3	28.5
TH+ DAT- Aldh1A1-	3	112.9	11.7	101.9	11.3	135.0	12.9

Table S18: Relative immunofluorescence signal intensities (RF) in SN DA neuron cellular compartments, determined manually and via DLAP-6. Data and statistics for graphs in Fig. 12b and S9 (background normalised). Number of total analysed neurons is given by n, N represents the number of analysed mice. P-values according to Kruskal-Wallis with Dunn's multiple comparison and Mann-Whitney test (significant values in bold).

TH [RF]										
		manual				automated				p-value
ROI	n	median	10-90 %	mean	±SD	median	10-90 %	mean	±SD	
cell body	94	272.8	125.6-534.1	307.9	145.4	284.4	134.4-542.0	317.8	147.3	0.6029
plasma-membrane		181.2	81.44-357.1	203.4	101.8	162.0	79.78-316.2	180.3	86.10	0.1707
nucleus		39.52	24.33-75.87	45.78	22.51	46.93	26.80-88.50	52.00	24.14	**0.0334**
p-values	Kruskal-Wallis: M; cell body vs nucleus, **<0.0001**; cell body vs membrane, **<0.0001**; A; cell body vs nucleus, **<0.0001**; cell body vs membrane, **<0.0001**;									
Kv4.3 [RF]										
		manual				automated				p-value
ROI	n	median	10-90 %	mean	±SD	median	10-90 %	mean	±SD	
cell body	94	2.3	1.4-3.8	2.5	1.0	2.3	1.3-3.9	2.6	1.1	0.5862
membrane		9.0	5.1-15.7	9.9	4.0	6.6	3.9-11.5	7.2	3.0	**<0.0001**
nucleus		0.6	0.3-1.2	0.6	0.4	0.6	0.3-1.1	0.7	0.4	0.9270
p-values	Kruskal-Wallis: M; membrane vs cell body, **<0.0001**; membrane vs nucleus, **<0.0001**; A; membrane vs cell body, **<0.0001**; membrane vs nucleus, **<0.0001**;									
DAPI [RF]										
		manual				automated				p-value
ROI	n	median	10-90 %	mean	±SD	median	10-90 %	mean	±SD	
cell body	94	1.9	1.0-3.2	2.1	1.1	1.6	0.9-2.8	1.8	0.9	0.0765
membrane		0.8	0.5-2.0	1.0	0.6	0.8	0.5-3.4	1.3	1.0	0.3500
nucleus		9.3	6.3-13.5	9.8	2.8	9.3	6.3-13.7	9.7	2.8	0.8212
p-values	Kruskal-Wallis: M; nucleus vs membrane, **<0.0001**; nucleus vs cell body, **<0.0001**; A; nucleus vs membrane, **<0.0001**; nucleus vs cell body, **<0.0001**;									

Table S19: Single cell correlation analyses between manually and DLAP-6 determined results. Data and statistics for graphs in Fig. 12c., according to Pearson correlation (slope of the linear regression with 95% confidence interval).

	Cytoplasm: TH	Plasma-membrane: Kv4.3	Nucleus: DAPI
Pearson r (95% confidence interval)	0.995 (0.992-0.997)	0.966 (0.949-0.977)	0.996 (0.994-0.008)
R^2	0.990	0.933	0.993
p-value	<0.0001	<0.0001	<0.0001
No. of XY pairs	94	94	94
Slope (95% confidence interval)	1.01 (0.989-1.029)	0.737 (0.696-0.777)	0.986 (0.969-1.00)
Proportionality constant ∝	0.96 ± 0.05	1.38 ± 0.15	1.01 ± 0.03

Table S20: Relative immunofluorescence-signal (RF) quantification for Kv4.3 in SN DA neuron membranes from WT and Kv4.3 KO mice, determined via DLAP-6. Data and statistics for graphs in Fig. 12e and S9 (background normalised). Number of analysed neurons is given by n, N represents the number of analysed mice. P-values according Mann-Whitney test (significant values in bold).

WT [RF]					Kv4.3 KO [RF]					p-value
n<br>N	median	10-90 %	mean	±SD	n<br>N	median	10-90 %	mean	±SD	
179	7.2	4.4-11.5	7.7	2.9	200	0.7	0.21-1.5	0.8	0.5	**<0.0001**
2	7.7	7.2-8.2	7.7	0.7	2	0.9	0.5-1.3	0.9	0.6	
