# Supplementary material for: Deep learning-based image analysis identifies a DAT-negative subpopulation of dopaminergic neurons in the lateral Substantia nigra
Source: Commun Biol. 2023 Nov 10;6:1146. doi: 10.1038/s42003-023-05441-6 (PMC10638391; doi:10.1038/s42003-023-05441-6)
Supplement: Supplementary file 3 — Description of Additional Supplementary Files [file 42003_2023_5441_MOESM3_ESM.docx]

**Description of Additional Supplementary Files**

**File name:** Figure S7

**Description:** Online interactive 3D-figure (HTML-file), plotting the anatomical location of all further analysed TH-positive SN neurons. a) Upper: sagittal (left) and coronal (right) mouse brain sections, modified from (Paxinos & Keith B. J. Franklin, 2007), illustrating the analysed caudo-rostral extent of the SN (bregma: -3.9 to -2.7, sagittal, blue), and its lateral parts in coronal sections (defined as >1.5 scaled x-units (>377.8 µm) lateral from each SN hemisphere-center (0,0); lateral SN: violet, non-lateral SN: grey, as in Figures 7-9, S6). Lower: plotted are the individual TH-positive SN neurons for all analysed animals (n = 38504, N = 14, as in Figure 7-9, S5), according to their scaled x,y,z-coordinates. The resulting anatomical 3D-representations display the medio-lateral distribution of the TH-positive DAT-negative SN neurons (violet) over the full rostro-caudal extent for all analysed mice. b/c) colour coded are the scaled relative DAT (b) and TH (c) signal intensities, of the individual TH-positive neurons from a), plotted according to their scaled x,y,z-coordinates. Colour coding for each neuron according to its individual deviation from the scaled mean signal-intensity (0.0) for each animal. The corresponding 2D maps are given in Figures 7-9.

**File name:** Supplementary Data 1

**Description:** Source data for all manuscript figures. Excel sheet tab name denotes the corresponding figures for which the underlying data is given.
