# Supplementary material for: Deep learning-based image analysis identifies a DAT-negative subpopulation of dopaminergic neurons in the lateral Substantia nigra
Source: Commun Biol. 2023 Nov 10;6:1146. doi: 10.1038/s42003-023-05441-6 (PMC10638391; doi:10.1038/s42003-023-05441-6)
Supplement: Supplementary file 5 — Figure S7 [file 42003_2023_5441_MOESM5_ESM.zip › Burkert_AI_online_supplement_20230928/Burkert_AI_figures_Online_supplement_20230808.html]

Online supplementary figure


# Online supplementary figure

## a) Localization of DAT- neurons

## b) DAT expression gradient

## c) TH expression gradient

**Figure S7: Online interactive 3D-figure plotting the
anatomical location of all further analyzed TH-positive SN
neurons.** a) Upper: sagittal (left) and coronal (right) mouse
brain sections, modified from (Paxinos & Keith B. J. Franklin,
2007), illustrating the analyzed caudo-rostral extent of the SN (bregma:
-3.9 to -2.7, sagittal, blue), and its lateral parts in coronal sections
(defined as >1.5 scaled x-units (>377.8 µm) lateral from each SN
hemisphere-center (0,0); lateral SN: violet, non-lateral SN: grey, as in
Figures 7-9, S6). Lower: Plotted are the individual TH-positive SN
neurons for all analyzed animals (n = 38504, N = 14, as in Figure 7-9,
S5), according to their scaled x,y,z-coordinates. The resulting
anatomical 3D-respresentations display the medio-lateral distribution of
the TH-positive DAT-negative SN neurons (violet) over the full
rostro-caudal extent for all analyzed mice. b/c) Color coded are the
scaled relative fluorescence-intensities of DAT (b) and TH (c) signals,
of the individual TH-positive neurons from a), plotted according to
their scaled x,y,z-coordinates. Color coding for each neuron according
to its individual deviation from the scaled mean signal-intensity (0.0)
for each animal. The corresponding 2D-maps are given in Figures 7-9.
